# Exome-first approach identified novel INDELs and gene deletions in Mowat-Wilson Syndrome patients

**DOI:** 10.1038/s41439-018-0021-y

**Published:** 2018-08-01

**Authors:** Maria Florencia Gosso, Cristian Rohr, Bianca Brun, Guadalupe Mejico, Fernanda Madeira, Fabian Fay, Melina Klurfan, Martin Vazquez

**Affiliations:** 1 Heritas - CIBIC S.A, Zeballos 249, Rosario, Argentina; 2 Heritas - INDEAR, Ocampo 210bis, Rosario, Argentina; 3 Casa Angelman, Esmeralda 280, Tigre, Buenos Aires Argentina

## Abstract

Mowat-Wilson syndrome (MWS) is characterized by severe intellectual disability, absent or impaired speech and microcephaly, with a gradual post-natal onset. The syndrome is often confused with other Angelman-like syndromes (ALS) during infancy, but in older children and adults, the characteristic facial gestalt of Mowat–Wilson syndrome allows it to be distinguished easily from ALS. We report two cases in which an exome-first approach of patients with MWS identified two novel deletions in the ZEB2 gene ranging from a 4 base deletion (case 1) to at least a 573 Kb deletion (case 2).

Mowat-Wilson Syndrome (MWS) is caused by haploinsufficiency of the *ZEB2* (ZFHX1B) gene on chromosome 2q22.3. MWS resembles Angelman Syndrome in that all individuals have moderate-to-severe intellectual disabilities and absent or impaired speech. Microcephaly, seizures and/or abnormal EEGs have been observed in up to 90% of affected individuals^1^. Affected people tend to have a smiling, open-mouthed expression and typically have friendly and happy personalities. During infancy, they are often misdiagnosed with other Angelman-like syndromes (ALS)^2, 3^; however, as they age, MWS patients begin to develop distinctive facial features, and adults with Mowat-Wilson syndrome have an elongated face with heavy eyebrows and a pronounced chin and jaw. The presence of congenital anomalies, including structural heart defects involving the pulmonary valve or arteries, hypospadias, and structural renal anomalies, also distinguishes MWS from ALS^4^. According to the Mowat-Wilson Foundation, there are currently 186 patients worldwide who have received genetic confirmation of the disease.

Since MWS is often misdiagnosed as another ALS during early infancy, it is very important to develop a first-tier single genetic test that covers all types of genetic mutations, including SNVs, INDELs and CNVs, to distinguish MWS from other ALS.

Whole Exome Sequencing (WES) or Clinical Exome Sequencing (CES) were both recently proposed for use in a first-tier diagnostic test for children with intellectual disabilities, as WES/CES has decreased costs compared to those of traditional diagnostic genetic tests^5^. The effectiveness of WES was also demonstrated for a wide variety of genetic disorders besides neurodevelopmental disorders for the detection of SNVs, INDELs, and CNVs using a single assay^6^. Here, we describe two MWS cases that were sent for genetic testing after being misdiagnosed with ALS for several years. The WES single assay allowed us to describe two novel mutations and to differentiate MWS unequivocally from ALS in both patients.

## Patient 1

A 17-year-old male from Misiones, Argentina who was born to healthy, non-consanguineous parents. After an uneventful pregnancy, he was referred for genetic testing after being diagnosed with ALS as an infant; he presented with a normal karyotype and CGH array test results. After years of being misdiagnosed, a genetic counselor suspected he might have been affected with MWS due to his facial features, congenital cardiomyopathy and the presence of generalized refractory epilepsy. He also presented with bilateral hearing loss, hypoplasia of the corpus callosum, and severe neurodevelopmental delay with the absence of speech.

## Patient 2

A 7-year-old male from Lobos in Buenos Aires, Argentina who was born to healthy, non-consanguineous parents. The relevant clinical features included a severe intellectual disability (ID), severe speech delay, and convulsive seizures. The patient presented with earlobe features that are characteristic of MWS. There was no reported family history of ID in the patient’s mother or in other known relatives. Previous testing included a 15p11.2-q13 methylation test, which was normal. This patient was initially diagnosed with ALS during infancy, when the typical phenotypic features were not clearly present^2^.

Blood samples were extracted after informed consent was obtained from the parents of each patient. DNA was then extracted from the blood samples using the High Pure PCR template purification kit (Roche S.A.Q.EI, Buenos Aires, Argentina) according to the manufacturer’s instructions. The DNA quality and concentration were assessed using an Implen NanoPhotometer (Biosystems SA, CABA, Argentina).

Next generation sequencing (NGS) of the whole exomes of each of the subjects was conducted according as follows. Prior to the preparation of the libraries, the DNA quality was assessed using a 2100 Bioanalyzer DNA chip (Analytical Technologies SA, Buenos Aires, Argentina). The samples were prepared using the Nextera Rapid Capture Exome Sequencing panel (Illumina, San Diego, USA). The libraries were sequenced with a NextSeq 500 System (Illumina, San Diego, USA) using a high-throughput kit and a configuration with a read length of 2 × 150 base pairs (bp) and dual indexing. All of the exomes were sequenced with 160 × coverage, with at least 93% of the sequences having a minimum of 20 × coverage.

To identify the germ-line variants present within the NGS data, which consisted of the sequences of the exons within the ZEB2 gene *(GRCh37/hg19 chr2:g*.145141048:145282747) and the adjacent intronic regions ( ± 10 bp), a proprietary bioinformatics analysis was performed that utilized a protocol based on that of the GATK (Genome Analysis Toolkit) from the Broad Institute. We included all variants with a minor allele frequency of at least 20% and with at least 4 reads that represented the alternative allele in our analysis. These variants were subject to comparison with entries in several databases and to analysis using in-sílico prediction programs. The classification of the variants was made according to guidelines published by the American College of Medical Genetics and Genomics (ACMG)^7^. The Copy Number Variants (CNVs) that were identified from the WES data were verified by a comparative genomics hybridization (CGH) array using an Innoscan710 instrument (Innopsis, Santa Clara, CA, USA) according to the manufacturer’s instructions.

**Fig. 1:**
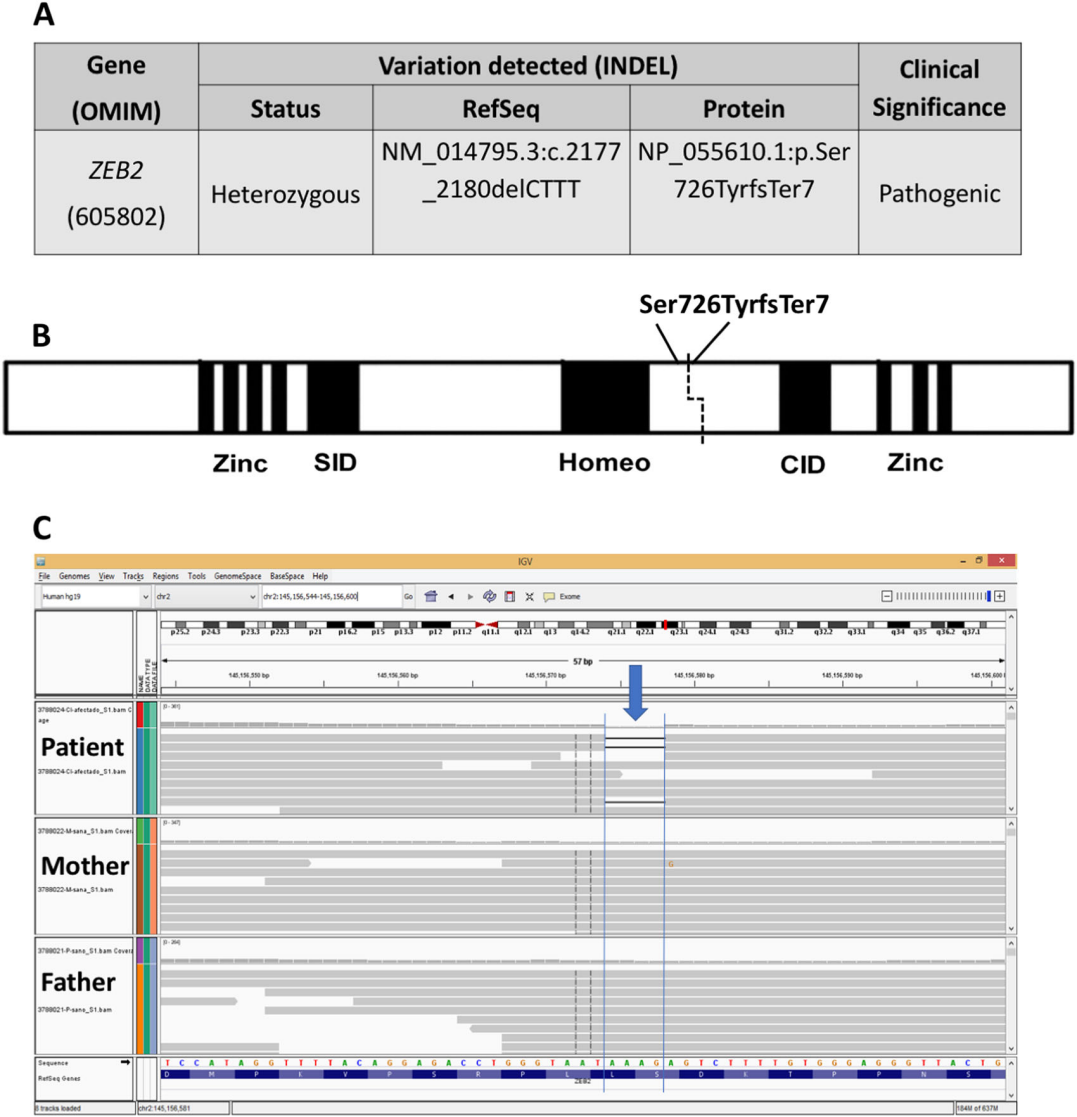
Patient 1 exome analysis. **a** The 4-bp INDEL variant of the *ZEB2* gene was identified in patient 1 from WES analysis. **b** Schematic representation of the *ZEB2* protein and its domains, indicating the location of the frameshift mutation that results in premature protein termination. **c** Screen capture of the BAM files analysis using the Integrative Genomics Viewer (http://software.broadinstitute.org/software/igv/bam) for the *ZEB2* INDEL p.Ser726TyrfsTer7 variant that was found in patient 1 and both of their parents. The blue arrow indicates the exact location of the 4-bp INDEL leading to a frameshift that results in premature protein termination

After obtaining the WES data for both patients as well as the parents of patient 1, the subsequent analysis identified a novel truncation variant (NM_014795.3:c.2177_2180delCTTT, NP_055610.1:p.Ser726TyrfsTer7) in patient 1 that was determined to be a deleterious mutation according to the variant interpretation guidelines of the ACMG (Fig. 1). This variant was not present in unrelated healthy controls that were obtained from the exome sequence databases ExAc Browser (http://exac.broadinstitute.org/) and gnomAD Browser (http://gnomad.broadinstitute.org/)^8^. The novel 4-bp INDEL that leads to the frameshift p.Ser726TyrfsTer7 (Fig. 1) was confirmed as de novo using Sanger sequencing^9, 10^. No other mutations were found in other genes in patient 1 that are known to be associated with ALS^2^, confirming the diagnosis of MWS and putting an end to years of misdiagnosis. However, no pathogenic mutations, including SNVs or INDELs, were identified in patient 2 in either *ZEB2* or other genes associated with ALS.

**Fig. 2:**
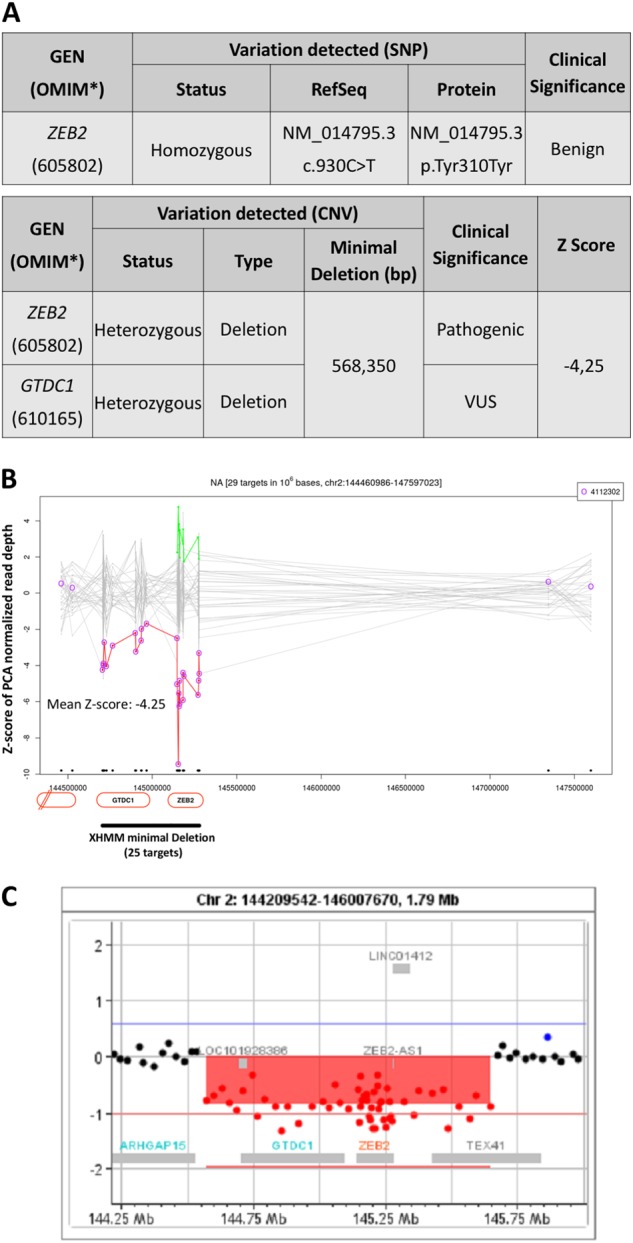
Patient 2 exome analysis. **a** Summary of the annotated SNVs in the *ZEB2* gene that were found to be either benign or pathogenic (8ZEB2 and VUS (GTDC1)) in the patient. **b** A detailed diagram of the deleted region (chr2:144704611-145277958) encompassing *ZEB2* as well as the neighboring *GTDC1* and *TEX41* genes as detected using the XHMM algorithm, which encompasses 25 target regions that are highlighted in red and are equivalent to 0.573 Mb. **c** The *ZEB2* pathogenic deletion (1.08 Mb), as confirmed by a CGH assay (arr[GRCh37] 2q22.3(144569168_145648045)x1), which includes the 0.573 Mb deletion detected via WGS. The CGH array extended the deleted region to include a partial deletion of the downstream *TEX41* gene, which is a non-protein coding gene of unknown function

The WES data for both patients were also screened for possible CNVs using a proprietary bioinformatics analysis protocol based on the eXome-Hidden Markov Model v1.0 (XHMM; https://atgu.mgh.harvard.edu/xhmm/)^11^. The screening analysis of CNVs in patient 2 identified a novel deletion of at least 0.573 Mb (*GRCh37/hg19* chr2:g.144704611-145277958) that was predicted to lead to the complete loss of the *ZEB2* gene, resulting in haploinsufficiency (Fig. 2a, b). This mutation was confirmed by a CGH array to be a novel 1.08 Mb heterozygous deletion (*arr[GRCh37] 2q22.3(144569168_145648045)x1*) that encompasses the 0.568 Mb deletion that was detected during the WES data analysis (Fig. 2c). The deleted region also encompasses the neighboring genes GTDC1, which is unrelated to MWS^12^, and TEX41, which is a non-protein coding gene of unknown function (http://www.genecards.org/cgi-bin/carddisp.pl?gene=TEX41). This deletion is not present in the Human Gene Mutation Database, version 2016.2 (HGMD; http://www.hgmd.org/), or in ClinVar (http://www.ncbi.nml.nih.org/clinvar/), which suggests that it is a novel pathogenic variant. Two other larger deletions encompassing the same region as this variant but with different breakpoints, ID 2566 (4.30 Mb) and ID 251811 (2.65 Mb), were found in the Decipher database (http://decipher.sanger.ac.uk). Inheritance of the deletion by patient 2 cannot be excluded, as a DNA sample from the father was not available, but it is reasonable to assume that it is a de novo deletion due to its pathogenic classification and the absence of any phenotypic features of MWS in either of the parents. Our genomic analysis confirmed suspicions of MWS in patient 2, despite an initial misdiagnosis of ALS. In contrast, no CNVs were identified from the WES data obtained from patient 1.

In summary, WES was performed on genomic DNA extracted from blood samples obtained from two patients and their parents, when available, after informed consent was obtained. Subsequent analyses revealed the presence of a novel pathogenic truncation variant (NP_055610.1: p.Ser726TyrfsTer7) of the *ZEB2 gene* in patient 1, which led to the loss of the CID and Zinc-finger 2 protein domains and resulted in haploinsufficiency (Fig. 1). Interestingly, in patient 2, no disease-causing SNVs or INDELs were identified; however, we were able to detect a heterozygous deletion of the entire *ZEB2* gene due to a large-scale deletion of the region encompassing *GRCh37/hg19* chr2:g.144704611-145277958. We were able to verify this deletion using a CGH array, which detected the presence of a larger chromosomal deletion (*arr[GRCh37] 2q22.3(144569168_145648045)x1*) that encompassed the deleted region that was identified via WES. The confirmed heterozygous deletion of a portion of chromosome 2 encompasses a gene that is upstream of *ZEB2*, *GTDC1* (MIM 610165), which encodes a glycosyltransferase-like domain-containing protein 1, as well as a downstream gene, *TEX41*, that does not encode a known protein and is of unknown function. As the Illumina Exome Capture kit does not screen for the *TEX41* gene, it was not detected during the WES CNV analysis. No association was reported in the literature between the large-scale *GTDC1-TEX41* deletion and any pathogenic phenotypes; thus, it is not possible to speculate whether these genes contribute to additional phenotypes in this patient other than those associated with MWS (http://www.genecards.org/cgi-bin/carddisp.pl?gene=TEX41)^13^. It is of note that we were able to correctly re-diagnose these patients as having MWS after both were misdiagnosed during infancy with ALS because the phenotypic features of MWS were not yet present.

The successful use of WES/CES as a first-tier single assay test for MWS has recently been highlighted as evidence that it can also be used for differential diagnosis of a wide variety of neurodevelopmental disorders. Using WES as a first-tier test in two patients with an early clinical diagnosis of ALS and normal 15p11.2-q13 methylation test results, we were able to identify two novel mutations that have not been previously described (a 4 bp deletion and *a* > 0.573 Mb deletion) that unequivocally differentiate MWS from other ALS. As a result, we propose that WES/CES can be used as a cost-effective first-tier assay to diagnose and differentiate MWS from ALS, which is caused by SNVs, INDELs, CNVs, or other factors, in newborns, infants, and young children with suspected ALS who also have a normal 15p11.2-q13 methylation test result.

## Data Availability

The relevant data from this Data Report are hosted at the Human Genome Variation Database at 10.6084/m9.figshare.hgv.2357 10.6084/m9.figshare.hgv.2360

## References

[1] Garavelli L, Mainardi PC (2007). Mowat-Wilson syndrome. Orphanet J. Rare Dis..

[2] Tan WH, Bird LM, Thibert RL, Williams CA (2014). If not Angelman, what is it? A review of Angelman-like syndromes. Am. J. Med. Genet. A.

[3] Luk HM (2016). Angelman-like syndrome: a genetic approach to diagnosis with illustrative cases. Case Rep. Genet..

[4] Garavelli L (2009). Mowat-Wilson syndrome: facial phenotype changing with age: study of 19 Italian patients and review of the literature. Am. J. Med. Genet. A.

[5] Monroe (2016). Effectiveness of whole-exome sequencing and costs of the traditional diagnostic trajectory in children with intellectual disability. Genet. Med..

[6] Pfundt (2017). Detection of clinically relevant copy-number variants by exome sequencing in a large cohort of genetic disorders. Genet. Med..

[7] Richards ycols (2015). 2015 Standards and guidelines for the interpretation of sequence variants: a joint consensus recommendation of the American College of Medical Genetics and Genomics and the Association for Molecular Pathology. Genet. Med..

[8] Karczewski KJ (2017). The ExAC browser: displaying reference data information from over 60 000 exomes. Nucleic Acids Res..

[9] Ivanovski I., et al. Phenotype and genotype of 87 patients with Mowat-Wilson syndrome and recommendations for care. Genet Med. 2018. 10.1038/gim.2017.221. [Epub ahead of print] PubMed PMID: 29300384. 10.1038/gim.2017.221 29300384

[10] Yamada Y (2014). The spectrum of ZEB2 mutations causing the Mowat-Wilson syndrome in Japanese populations. Am. J. Med. Genet. A.

[11] Fromer M, Purcell SM (2014). Using XHMM software to detect copy number variation in whole-exome sequencing data. Curr. Protoc. Hum. Genet..

[12] Zhao E (2004). Cloning and expression of human GTDC1 gene (glycosyltransferase-like domain containing 1) from human fetal library. DNA Cell Biol..

[13] Park JY (2013). Mowat-Wilson syndrome detected by using high resolution microarray. Gene.

